# Forced fluid removal versus usual care in intensive care patients with high-risk acute kidney injury and severe fluid overload (FFAKI): study protocol for a randomised controlled pilot trial

**DOI:** 10.1186/s13063-017-1935-2

**Published:** 2017-04-24

**Authors:** Rasmus E. Berthelsen, Theis Itenov, Anders Perner, Jens-Ulrik Jensen, Michael Ibsen, Andreas Emil Kryger Jensen, Morten Bestle

**Affiliations:** 1 Department of Intensive Care, Nordsjællands Hospital, Hillerød, Denmark; 2 Department of Intensive Care, Rigshospitalet, Copenhagen, Denmark; 3 CHIP & PERSIMUNE, Department of Infectious Diseases, Rigshospitalet, Copenhagen, Denmark; 4 Department of Public Health, Copenhagen University, Copenhagen, Denmark

**Keywords:** Acute kidney injury, Fluid, Fluid overload, Intensive care, Randomised trial, Feasibility trial

## Abstract

**Background:**

Intravenous administration of fluids is an essential part of critical care. While some fluid administration is likely beneficial, there is increasing observational evidence that the development of fluid overload is associated with increased mortality. There are no randomised trials to confirm this association in patients with acute kidney injury. We aim to perform a pilot trial to test the feasibility of forced fluid removal compared to standard care in patients with acute kidney injury and severe fluid overload, the FFAKI trial.

**Methods:**

Then FFAKI trial is a pilot, multicentre, randomised clinical trial recruiting adult intensive care patients with acute kidney injury and fluid overload, defined as more than 10% of ideal bodyweight. Patients are randomised with concealed allocation to either standard care or forced fluid removal with a therapeutic target of negative net fluid balance ≥1 mL/kg/h. The safety of fluid removal is continually evaluated according to predefined criteria of hypoperfusion: lactate ≥4 mmol/L, mean arterial pressure <50 mmHg or mottling beyond the edge of the kneecaps. If patients fulfil one of these criteria, fluid removal is suspended until hypoperfusion has resolved. The primary outcome measure is fluid balance at 5 days after randomisation and secondary outcomes include mean daily fluid balance, fluid balance at discharge from the intensive care unit, time to neutral fluid balance, number of serious adverse reactions and number of protocol violations. All patients are followed for 90 days.

**Discussion:**

The FFAKI trial started in October 2015 and, when completed, will provide data to evaluate whether a large trial of forced fluid removal in critically ill patients is feasible. Our primary outcome will show if the experimental intervention leads to a clinically relevant difference in fluid balance, which could prove beneficial in intensive care patients with acute kidney injury.

**Trial registration:**

EudraCT, identifier: 2015-001701-13. Registered on 19 September 2015;

ClinicalTrials.gov, identifier: NCT02458157. Registered on 21 May 2015;

Danish Ethics Committee, identifier: H-15009589H. Registered on 22 September 2015; Danish Health and Medicines Authority, identifier: 2015070013. Registered on 11 August 2015.

**Electronic supplementary material:**

The online version of this article (doi:10.1186/s13063-017-1935-2) contains supplementary material, which is available to authorized users.

## Background

Administration of isotonic crystalloid solutions is a common intervention in critically ill patients. In some cases this leads to the accumulation of fluids and development of fluid overload, defined as a positive fluid balance corresponding to 10% or more of total bodyweight. The cut-off at 10% was first utilised in a paediatric observational study by Gillespie et al. [1], and subsequently adopted in the adult population [2]. Growing observational data have linked fluid overload to a poor outcome in several different patient populations including those with acute kidney injury (AKI) [3]. This was analysed in a recent systematic review and meta-analysis of data from 12 cohort studies including 5095 patients [4]. Six of the 12 studies reported adjusted odds ratios (ORs) for death, and the pooled results associated fluid overload with increased risk of death with an OR of 2.23 (95% CI, 1.66–3.01) and a moderate level of heterogeneity (*I* ^*2*^ = 62%). Mean positive daily fluid balance was also shown to be associated with death with an OR of 1.16 (95% CI, 1.07–1.27); however, there was significant heterogeneity amongst the studies (*n* = 6, *I* ^*2*^ = 94%).

The observed association between fluid overload and outcome may in part be mediated by the development of interstitial oedema leading to deranged organ architecture, increased diffusion distances for oxygen and metabolites and increased interstitial pressure [5].

A study in healthy volunteers demonstrated that infusion of 2 L crystalloid leads to renal swelling [6]. Renal interstitial volume and pressure may be correlated in a nonlinear manner, suggesting that a ‘renal compartment’ exists [7] and increased interstitial pressure has been linked to a decline in renal blood flow (RBF), glomerular filtration rate (GFR) and sodium excretion [8, 9]. The detrimental effect of fluid overload has been attenuated in experimental renal decapsulation in both animals and humans [10].

Given the observational nature of the available data, there is a high risk of confounding by indication and a causal relationship cannot be established before randomised data are available [11]. We aim to perform a feasibility trial of forced fluid removal in intensive care unit (ICU) patients with AKI and severe fluid overload. If we are able to achieve a clinically relevant difference in fluid balance we believe that a definitive trial powered for mortality is warranted.

## Methods

The FFAKI trial is a multicentre, randomised, site-stratified, clinical pilot trial with adequate computer generation of the allocation sequence with permuted blocks of varying size and allocation concealment. Randomisation is performed using sequentially numbered, opaque, sealed envelopes.

The trial statistician is blinded but due to the nature of the intervention it is not possible to blind patients or caregivers.

The trial is initiated at three separate centres in Denmark. Each centre has one or two primary investigators with expert knowledge in the FFAKI intervention and protocol. Participants in the FFAKI trial will receive protocol-specific treatment during their entire ICU stay. Therefore, every caregiver at participating centres receives training in the protocol by either the principal investigator or one of the primary investigators. It is expected that the primary centre (Nordsjællands Hospital) will include 20–30 patients and each secondary centre will include 10–15 patients.

**Fig. 1:**
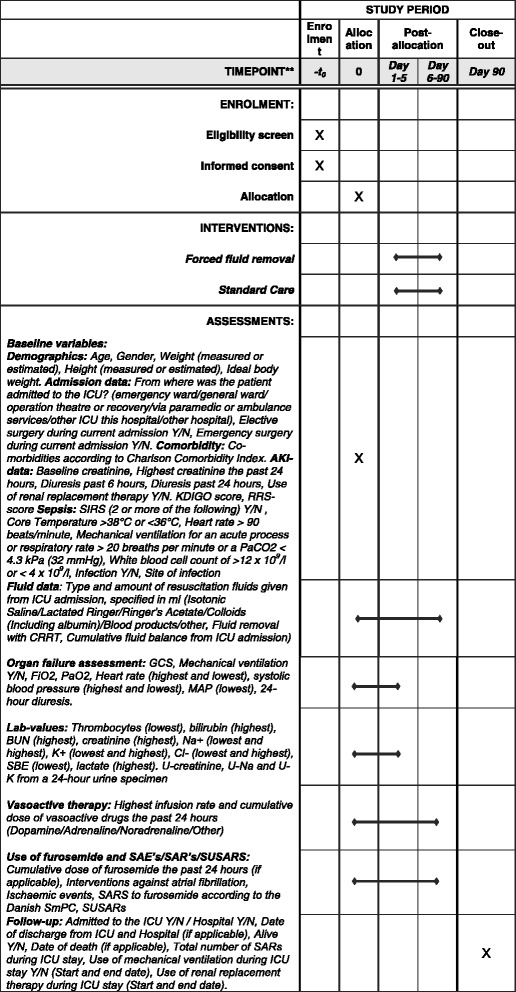
Standard Protocol Items: Recommendations for Interventional Trials (SPIRIT) figure for the FFAKI trial. CONSORT 2010 Flow Diagram for the FFAKI-trial

The trial protocol was written according to the Standard Protocol Items: Recommendations for Interventional Trials (SPIRIT) Statement [12]. A populated SPIRIT Checklist and figure are provided in Additional file 1 and Fig. 1, respectively.

### Patients

All patients admitted to the ICU at the participating centres will be eligible for screening according to the following:

#### Inclusion criteria

- Age 18 years or older
- AKI defined according to the Kidney Disease Improving Global Outcomes (KDIGO) criteria [13]
- Renal Recovery Score (RRS) ≤60%
- Fluid overload defined as a positive fluid balance of at least 10% of ideal body weight
- Able to undergo randomisation within 12 h of fulfilling the other inclusion criteria

#### Exclusion criteria

- Known allergy to furosemide or sulphonamides
- Known prehospitalisation advanced chronic kidney disease (estimated glomerular filtration rate (eGFR) <30 mL/min/1.73 m^2^ or chronic renal replacement therapy)
- Severe hypoxic respiratory failure (use of invasive ventilation and fraction of inspired oxygen (FiO_2_) >80% and positive end-expiratory pressure (PEEP) >10 cmH_2_O)
- Severe burn injury (≥10% total burned surface area)
- Severe dysnatraemia (plasma concentrations <120 or >155 mmol/L)
- Hepatic coma
- Mentally disabled undergoing forced treatment
- Pregnancy/breastfeeding
- Lack of commitment for ongoing life support including renal replacement therapy (RRT)
- Lack of informed consent

**Fig. 2:**
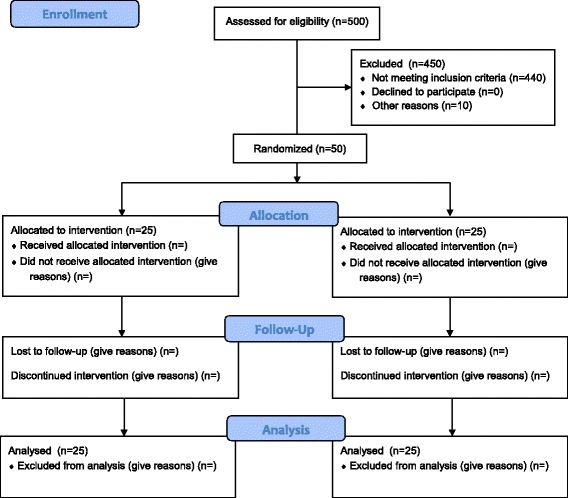
Consolidated Standards of Reporting Trials (CONSORT) diagram

Patients who fulfil all of the inclusion criteria and none of the exclusion criteria will be enrolled in the trial and allocated to either the experimental treatment (forced fluid removal) or standard care. Based upon own observational data we expect that roughly 10% of adult patients admitted for more than 24 h will be available for inclusion in the FFAKI trial (Fig. 2).

### Forced fluid removal (Figs. 3, 4 and 5)

**Fig. 3:**
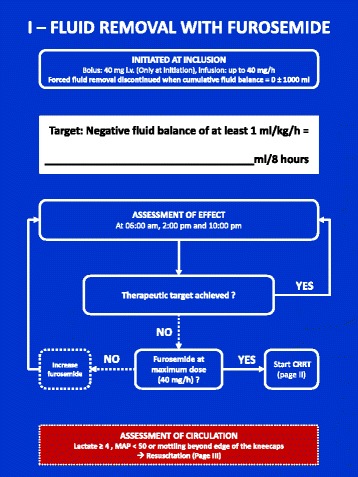
FFAKI algorithm for fluid removal with furosemide

**Fig. 4:**
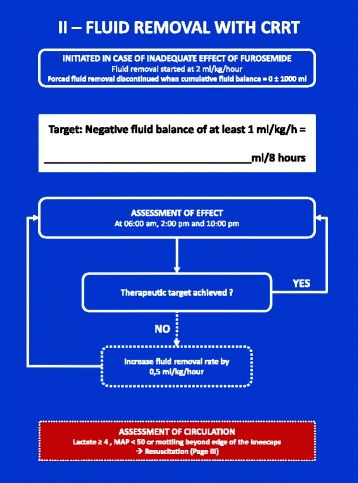
FFAKI algorithm for fluid removal with continuous renal replacement therapy (CRRT)

**Fig. 5:**
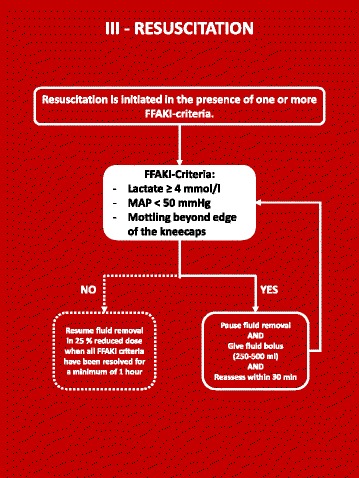
FFAKI resuscitation algorithm

The experimental intervention is guided by a therapeutic goal of average net negative fluid balance ≥1 mL/kg/h and safety variables indicating inadequate circulation (lactate ≥4 mmol/L or mean arterial pressure (MAP) <50 mmHg or mottling beyond the edge of the kneecaps).

The therapeutic effect is evaluated at three daily summary points on the ICU observation charts (06:00, 14:00 and 22:00), while safety variables are evaluated continuously.

The first choice for fluid removal is diuretic therapy with furosemide which is continued for a minimum of 8 h. If the therapeutic goal cannot be achieved and maintained by diuretic therapy it is replaced by fluid removal with continuous renal replacement therapy (CRRT).

### Resuscitation

During the entire trial, the physiologic response to fluid removal is monitored with three variables indicating inadequate circulation. These are:

- Mottling beyond the edge of the kneecaps [14]
- Hypotension (MAP <50 mmHg) resistant to inotropes and vasopressors
- Plasma lactate ≥4 mmol/L [15]

Mottling and MAP are monitored continuously and lactate is routinely measured four to six times each day and on clinical indication.

If one or more signs of inadequate circulation are present the resuscitation algorithm is started:

- Pause fluid removal
- Give a crystalloid fluid bolus of 250–500 mL
- Re-evaluate circulatory status within 30 min
- Repeat fluid therapy and revaluation until adequate circulation (lactate <4 mmol/L, MAP >50 mmHg and no mottling beyond kneecaps) have been maintained for minimum 1 h
- Restart fluid removal in 25% reduced dose for a minimum of 4 h before evaluating effect

Fluid removal is continued until the patient achieves neutral cumulative fluid balance (±1000 mL), which is then attempted maintained for the entire duration of ICU admission.

### Standard care

In the control group receiving standard care, there are no protocolled interventions. Fluid administration and removal is done at the discretion of the treating clinicians. Use of dialysis is encouraged only in the presence of severe disturbances in fluid, electrolyte and acid-base balance:

- Hyperkalaemia (p-K^+^ >6 mmol/L)
- Severe metabolic acidosis attributable to AKI (pH <7.25 and standard base excess (SBE) below −10 mmol/L) resistant to intravenously (IV) administered bicarbonate infusion
- Severe respiratory failure with PaO^2^/FiO^2^ < 13 kPa and bilateral infiltrates/oedema on the chest X-ray
- Aside from these absolute indications, RRT may be provided in case of progressive azotaemia and blood urea (BUN) >25 mmol/L

All data are collected in a paper-based Case Research Form (CRF) and participants are followed for a total of 90 days. The primary outcome of the FFAKI trial is cumulative fluid balance 5 days after randomisation. All outcome variables are shown in Table 1. Furthermore, we will evaluate the safety of the intervention by registering known serious adverse reactions (SARs) to furosemide and serious adverse events (SAEs) to fluid removal including: arrhythmia, ischaemia, vasopressor use and progression of organ failure as captured in the daily Sequential Organ Failure Score (SOFA) score.

**Table 1:** Primary, secondary and exploratory outcomes for the FFAKI trial

Primary outcome	Cumulative fluid balance 5 days after randomisation
Secondary outcomes	Mean daily fluid balance during ICU stay
	Cumulative fluid balance during the entire ICU stay
	Time to neutral cumulative fluid balance
	Number of patients with one or more major protocol violations
	Accumulated SARs in each intervention arm during the ICU stay
Exploratory outcomes	All-cause mortality at day 90
	Days alive and out of hospital within 90 days of follow-up
	Days alive without mechanical ventilation within 90 days of follow-up
	Days alive without vasopressor/inotropic therapy within 90 days follow-up
	Days alive without RRT within 90 days follow-up
	Renal recovery at day 90

*ICU* intensive care unit, *RRT* renal replacement therapy, *SAR* serious adverse reaction

### Monitoring and protocol adhesion

The FFAKI trial is externally monitored according to Good Clinical Practice (GCP) (EU-Directive-2001/20) guidelines including monitoring of consents and source data by external staff.

Protocol adhesion is monitored by the GCP guidelines on the first five patients in both the intervention and control arm at each centre and protocol violations are registered in the CRF of all patients. In case of protocol violations the principle investigator will initiate re-education of participating caregivers.

### Statistical analysis

The main objective of the FFAKI trial is to determine whether the trial intervention is feasible. To capture this we have decided to examine whether the trial intervention leads to a clinically relevant difference in fluid balance after 5 days. This is treated as an interim outcome and in accordance with the newly published Consolidated Standards of Reporting Trials (CONSORT) extension to randomised pilot and feasibility trials [16], we used the standard method of calculating sample size. By including 50 patients in the trial we will have the power to show a difference of 6 L in cumulative fluid balance between groups with a *β* value of 0.80 and a two-sided *α* value of 0.05. The sample size estimation is based upon observational data of patients fulfilling the FFAKI inclusion criteria admitted at our ICU in 2012 and 2013 (mean fluid balance 13.8 L, standard deviation (SD) 7.4 L).

Two complimentary analyses of the primary outcome will be performed in order to account for attrition due to death. In the first analysis the subject-specific fluid balances are modelled in a ‘linear random-effects model’ unconditional on survival status. Difference in fluid balance 5 days after randomisation will be assessed using a Wald test. In the second analysis we will estimate the ‘survival average causal effect’ of the intervention using principal stratification and include a sensitivity analysis to assess the influence of possible violations of assumptions. In both models missing data due to dropout will be handled using ‘inverse probability weighting’.

All parametric data will be presented as mean (SD) and compared using Student’s *t* test. Nonparametric data will be reported as median (interquartile range (IQR)) and compared using the Mann-Whitney *U* test.

## Discussion

The FFAKI trial consists of a complex intervention that alters the core of current therapy for critically ill patients. Furthermore, the intervention is designed to treat an iatrogenic condition (fluid overload). To our knowledge there are no previous implementations of forced fluid removal in a randomised clinical design and we have identified several key areas in which a trial of forced fluid removal might prove unfeasible:

1. Patient physiology might not allow early and forced fluid removal
  Patients who develop AKI and fluid overload are characterised by severe critical illness, often with failure of multiple organ systems and they receive a multitude of interventions including mechanical ventilation, fluid therapy, antibiotics, vasopressors, inotropes and dialysis. The underlying condition further compromises the physiology of the patient leading to leaky capillaries and loss of intravascular albumin [17]. Fluid removal by diuretics or CRRT is done from the intravascular compartment and the extracorporeal removal of fluids depends upon the compensatory movement of fluids from the extravascular compartment to the intravascular compartment [11]. These factors might oppose removal of fluids and patients who undergo forced fluid removal could develop further worsening of circulatory status leading to discontinuation of fluid removal according to the safety parameters described earlier. Previous trials have suggested that fluid restriction and fluid removal in critical illness is safe and well tolerated by patients admitted to the ICU. The FACCT trial [18] showed a difference in fluid balance of 7 L with restricted fluid therapy in patients with acute lung injury. Ganter and co-workers performed fluid removal in 10 critically ill patients with a mixed medical history [19] and achieved very large cumulative volumes of fluid removal ranging from 7.4 to 19.8 L during the course of 72 h

2. The Hawthorne effect might alter the administration of IV fluids leading to reduced incidence of fluid overload and abolish the need for forced fluid removal
  Fluid overload is an iatrogenic condition that arises as a consequence of the current practice of fluid therapy in critical illness. When a trial of forced fluid removal is initiated there will be an inevitable increased focus on the indications for fluid administration and removal. This produces a risk of a Hawthorne effect leading to less fluid administered by clinicians which would reduce the incidence of fluid overload in the given patient population and abolish the need for a trial of this nature

3. Heterogeneity of the patient population could dilute any potential effect of the intervention leading to reduced power and the need for a very large sample size
  The baseline chance of recovering renal function following AKI in the ICU varies with age, gender and the severity of kidney injury. Patients who have a high chance of recovering with the current standard of care will have very little potential benefit of participating in a trial of new therapeutic interventions. Furthermore, these patients could dilute the potential effect of a given intervention and lead to a loss in power and need for larger sample sizes. We have recently developed a model to predict the chance of recovering renal function following AKI in the ICU, which is called the ‘Renal Recovery Score’ (RRS) (not yet published). In the development and validation of the model we found that the 20% of patients with the highest chance of recovering renal function had a RRS value of >64% and an 80–85% frequency of recovery. To selectively include patients with a moderate to high risk of persistent renal injury we will be using a RRS value of <60% as part of the inclusion criteria

4. Lack of blinding
  Due to the nature of the intervention it is not possible to blind participants, caregivers administering the intervention or caregivers administering co-interventions. This introduces a risk of bias which we will evaluate with fluid therapy and dialysis data in both treatment arms. To avoid overly aggressive fluid removal in the control group we discourage the use of dialysis unless specifically indicated according to previously described criteria

5. Lack of protocol adherence
  Forced fluid removal in critically ill patients with continued need for vasopressors and inotropes might seem controversial to some clinicians and it is uncertain whether our intervention can be performed with the acceptance of the treating clinicians. Therefore, we register the frequency of protocol violations defined as the use of dialysis outside the recommended indications in the control group, and cessation of fluid removal before fulfilment of safety criteria or achievement of neutral fluid balance in the experimental group

By performing a pilot trial with a primary outcome of fluid balance 5 days after randomisation we will be able to evaluate whether the intervention is feasible. Furthermore we will know if it leads to a clinically relevant difference in fluid balance which would indicate that implementation of this protocol in a larger sample size would provide analysable results regarding a causal relationship between fluid removal and patient-centred outcomes of renal recovery and survival.

## Trial status

The FFAKI trial was initiated in October 2015 and recruitment is currently ongoing at three different sites.

## Additional file

Additional file 1: Populated SPIRIT Checklist. (PDF 130 kb)

## Abbreviations

AKI: Acute kidney injury
BUN: Blood urea nitrogen
CI: Confidence interval
CRF: Case Research Form
CRRT: Continuous renal replacement therapy
eGFR: Estimated glomerular filtration rate
FFAKI: Forced fluid removal versus usual care in intensive care patients with high-risk acute kidney injury and severe fluid overload
FiO_2_: Fraction of inspired oxygen
GCP: Good Clinical Practice
GFR: Glomerular filtration rate
ICU: Intensive care unit
IQR: Interquartile range
IV: Intravenous
KDIGO: Kidney Disease Improving Global Outcomes
MAP: Mean arterial pressure
OR: Odds ratio
PEEP: Positive end-expiratory pressure
RBF: Renal blood blow
RRS: Renal Recovery Score
SAE: Serious adverse event
SAR: Serious adverse reaction
SBE: Standard base excess
SD: Standard deviation
SOFA: Sequential Organ Failure Score
